# Benefits of Mindfulness for Parenting in Mothers of Preschoolers in Chile

**DOI:** 10.3389/fpsyg.2018.01443

**Published:** 2018-08-17

**Authors:** Carolina Corthorn

**Affiliations:** Faculty of Education, Universidad Andrés Bello, Santiago, Chile

**Keywords:** DASS-21, mindfulness, parental stress, parenting, preschoolers

## Abstract

The present study evaluated whether mothers’ participation in a mindfulness-based intervention led to statistically significant differences in their general levels of stress, depression, anxiety, parental stress, mindful parenting, and mindfulness. Forty-three mothers of preschool-age children participated, 21 in the intervention group and 22 in the comparison group. Scores of mental health variables were within normal ranges before the intervention. All of the participants worked at the *Universidad Católica de Chile* (Catholic University of Chile), and their children attended university preschool centers. Repeated measured ANOVA analysis were performed considering differences between gain scores of each group, rather than post-treatment group differences. This was chosen in order to approach initial differences in some of the measures (mindfulness, mindful parenting, and stress) probably due to self-selection. As predicted, the intervention group showed a significant reduction in general and parental stress and an increase in mindful parenting and general mindfulness variables when compared with the comparison group. Effect sizes ranged from small to medium, with the highest Cohen’s *d* in stress (general and parental) and mindful parenting. In most cases, the significant change was observed between pre- and post-test measures. Follow-up measures indicated that the effects were maintained after 2 months.

## Introduction

It is well known that parenting and parent–child relationships influence multiple aspects of children’s social and emotional development (Bowlby, 1988; NICHD, 2005; Waylen et al., 2008; Marrone, 2014). In this context, because of the abundant evidence regarding the benefits of mindfulness (see reviews: Baer, 2003; Grossman et al., 2004; Greeson, 2009; Hofmann et al., 2010), there has been increased interest in incorporating mindfulness-based strategies into parenting intervention (e.g., Dumas, 2005; Duncan et al., 2009).

The practice of mindfulness, defined as an awareness that arises through “paying attention in a particular way, on purpose, in the present moment, non-judgmentally” (Kabat-Zinn, 2003), has been found to significantly reduce stress, anxiety, and depression (Baer, 2003; Hofmann et al., 2010); to increase positive emotions; and to improve overall quality of life (Brown et al., 2007; Greeson, 2009). Several studies have also reported effects on relationship aspects such as openness, ability to relate, intimacy, emotion regulation (Brown and Ryan, 2003; Brown et al., 2007) identification, communication of feelings, anger management (Wachs and Cordova, 2007) and empathetic response (Beitel et al., 2005; Block-Lerner et al., 2007; Dekeiser et al., 2008).

Evidence that indicate the positive effects of mindfulness in the general population suggest that it could be relevant in parent–child relationships (e.g., Kabat-Zinn and Kabat-Zinn, 1997) and that including it in parenting programs could yield positive effects (Dumas, 2005). Several recent studies support this approach (Singh et al., 2006, 2007, 2010a,b; Altamaier and Maloney, 2007; Dawe and Harnett, 2007; Vieten and Astin, 2008; Coatsworth et al., 2010; Perez-Blasco et al., 2013; Bögels et al., 2014). How can mindfulness be helpful for parenting?

First, the evidence regarding reduction of stress, anxiety, and depressive symptoms would be enough reason for incorporating mindfulness in parenting interventions. Several empirical studies have found associations between higher levels of parental stress and less appropriate parent–child interactions, dysfunctional parenting, and behavioral problems in children (Webster-Stratton, 1990; Creasey and Jarvis, 1994; Sidebothan, 2001; Bonds et al., 2002). With regard to depression, there is strong support for the relationship between parental depressive symptoms and negative parenting (e.g., Stein et al., 1991; Lovejoy et al., 2000; Goodman and Gotlib, 2002; Restifo and Bögels, 2009; Quezada and Santelices, 2010). Additionally, parental history of mood and anxiety disorders is one of the strongest and most consistent risk factors for the development of these disorders in their children, even in non-clinical samples (Low et al., 2012).

Second, mindfulness dimensions as described in Baer et al.’s (2006) Five-Facet Mindfulness Questionnaire (FFMQ) could be beneficial for parenting: “acting with awareness,” “observing,” “describing,” “non-reactivity to inner experience,” and “non-judging of inner experience.” “Acting with awareness,” that is, being fully attentive and conscious of the experiences of the present moment, is considered to be essential for effective parenting because this implies that the parent is truly present in the moment-to-moment parent–child interactions and is therefore more able to emotionally connect with her child and respond to his or her needs (Duncan et al., 2009). “Observing” and “Describing” are mindfulness dimensions that imply being able to recognize and name your own thoughts, emotions, feelings and sensations. These aspects, -related to emotional intelligence (Baer et al., 2006) and emotional awareness, -are important for empathy and considered essential to good parenting (Kabat-Zinn and Kabat-Zinn, 1997; Barudy and Dantagnan, 2005). “Non-judging” implies accepting thoughts, feeling and experiences without evaluating or over-identifying with them. This aspect entails greater kindness toward the mother herself and acceptance of parental mistakes and the child as a human being. “Non-reactivity” refers to the self-regulation aspect of mindfulness. Mindfulness practice teaches parents to stop and be aware of their automatic tendencies before acting. In this way, negative automatic interactions in parenting may decrease.

Mindful parenting has been described as specific and differentiated from mindfulness in general. Kabat-Zinn and Kabat-Zinn (1997) define mindful parenting as “paying attention to your child and your parenting in a particular way: intentionally, here and now, and non-judgmentally.” Duncan et al. (2009) proposed a model of mindful parenting that encompasses five dimensions, derived from the more general mindfulness aspects: listening with full attention, non-judgmental acceptance of self and child, emotional awareness of self and child, self-regulation in parenting relationships and compassion for self and child.

The research on mindful parenting is increasing but it is still an emerging field and further evidence is definitely needed. Studying the results of mindfulness-based interventions for mothers can help give light regarding the possibilities of mindfulness practice as a way of generating positive change in parenting. It has been found that through participating in mindfulness-based interventions parents can reduce levels of stress and mood disorders (Singh et al., 2007; Vieten and Astin, 2008; Duncan and Bardacke, 2010; Bögels et al., 2013; Perez-Blasco et al., 2013), improve parental well-being, mindful parenting and parent–child interaction (Coatsworth et al., 2010, 2015), improve parenting, co-parenting, parental satisfaction and family functioning (Singh et al., 2006, 2007, 2010b; Duncan and Bardacke, 2010; Bögels et al., 2013), reduce potential of child maltreatment (Dawe and Harnett, 2007), and reduce hyperactivity symptoms (Van der Oord et al., 2012). There are also some studies that assess the relation among mindfulness in parents and other parenting variables, without intervention (MacDonald and Hastings, 2010; Parent et al., 2010; Williams and Wahler, 2010; Bluth and Wahler, 2011), finding that higher levels of mindfulness are associated with lower of depressive symptoms, higher involvement in parental tasks and roles associated to child socialization, lower amount of internalizing and externalizing behavior in their children, and authoritative parenting style, and reduced perceived level of effort required for parenting.

Only a few studies about mindful parenting are available that include comparison groups and follow-up measures (Bögels et al., 2008, 2010; Coatsworth et al., 2010, 2015; Perez-Blasco et al., 2013). For example, in a randomized trial study, Coatsworth et al. (2010) found that adding mindfulness to an already empirically validated parenting program improved mindful parenting variables and the quality of parent–child interaction.

Also, research is needed regarding mindful parenting on specific developmental stages. Studies focused on mothers of preschool age children are scarce. To the knowledge of the author only two have been published. One of them was a correlational study that evaluated parent’s effort related to parenting preschoolers (Bluth and Wahler, 2011) and the other evaluated pre–post intervention measures in a group of divorced parents of preschoolers (Altamaier and Maloney, 2007). Mindfulness practice is particularly relevant in the preschool years. At this age, due to a normal need of the child for autonomy, children usually present an increase of oppositionist behavior and frustration, often leading to temper tantrums. Also, the preschool child is expected to develop basic socialization skills, which usually include not having his or her desires fulfilled at will. Therefore, conflicts tend to arise more often around this developmental stage. During this stage, mindfulness practice would allow parents to be less stressed, to have less automatic reactions during the intense parent–child interactions, and to be a better model for the child to learn self-regulation.

Therefore, in an effort to contribute to scientifically based knowledge in this field of research, the present study focused on evaluating the effects of a mindful-based program in a group of highly educated mothers with regard to several relevant psychological variables (stress, depression, and anxiety) and their level of mindfully attending to their preschoolers. We will compare the results with mothers from similar background who did not participate in the program. It was predicted that, after participating in an 8-week mindfulness-based intervention for mothers, the participants, compared to the comparison group, would significantly increase levels of being mindful, as measured by Baer et al.’s (2006) FFMQ Scale, and mindful parenting, as measured by Duncan’s (2007) IM-P Scale in its Chilean adaptation Spanish version (Corthorn et al., 2015, unpublished), in the following sub-dimensions: Observe (FFMQ), Non-judge (FFMQ), Non-react (FFMQ), Non-judgmental acceptance of self as a mother (IM-P), Empathy and acceptance for the child (IM-P), and Self-regulation in the parenting relationship (IM-P). Additionally, that mothers from the intervention group would have significantly reduced levels of depression, anxiety, and stress (DASS), as measured by DASS-21 (Lovibond and Lovibond, 1995), and Parental Stress as measured by PSI-SF (Abidin, 1995). For the mindfulness sub-dimension “Act with awareness” and the mindful parenting sub-dimension “Listening with full attention,” it was predicted that mothers whose initial scores were lower would increase but that there may be decreases in scores of mothers who initially presented higher levels in these dimensions. The reason for this was that mothers with initially high scores would probably become more aware of self-distractions, thus responding in a way that would indicate lower results in these dimensions after de program. This would impede statistically significant results. Finally, it was expected that the mothers from the intervention group would not present an increase in FFMQ “Describe” sub-dimension because this aspect was less specifically addressed in the intervention.

## Materials and Methods

### Participants

Forty-three mothers of preschool children (2–5 years old) participated in this study, 21 in the intervention group and 22 in the comparison group. All of them worked at the *Pontificia Universidad Católica de Chile* (Catholic University of Chile) and their children attended one of three university preschool centers. The preschool centers are settled in university campuses located in different neighborhoods in Santiago, Chile. Regarding bias and blindness between groups, it is important to notice that Catholic University of Chile is a very big organization and participants’ workplaces were located in different locations around the university. Even so, there still could have been some risk of exposure to information between groups since it was not possible to control this aspect in the study. The average age of the mothers was 35.6 years old (*SD* = 5.2). Most of them were either married or living with their partners (72.1%), and they had an average of two children. A total of 16.3% of them were single mothers, and 11.6% were divorced or separated. They were mostly highly educated women, 60.5% of them had a university degree and 34.9% had a technical education. Most fathers of the children also had a university degree (55.8%) or a technical degree (27.9%). More than half of the participants reported monthly salaries above or within Santiago Metropolitan Region’s average, which is approximately US $2,000 (Instituto Nacional de Estadística [INE], 2013), with 44.2% of the families falling between US $1,800 and US $5,400 per month and 9.3% above US $5,400. Importantly, 46.5% of the participants of this study reported monthly family income lower than the regional average, some of them far below (14% between US $1,300 and $1,800, 20.9% between US $800 and $1,300, and 11.6% between US $400 and $800).

### Procedure

Mothers whose children were between 2 and 5 years old and attended any of the three preschool centers at *Pontificia Universidad Católica de Chile* were invited to an introductory talk. In this initial meeting, the program was explained in detail and questions and doubts were addressed, so mothers could decide whether they were interested in participating. They were informed about the study and that they would be asked to respond to several questionnaires, on three occasions: immediately before and after the intervention, and 2 months later for follow-up. This information was delivered verbally and through an informed consent letter. At the end of this meeting, women that were interested in participating completed the pre-intervention set of questionnaires. Mothers participating in the comparison group were contacted directly through the preschool center. It is important to notice that comparison group participants did not attend the introductory talk. They participated by answering the same questionnaires as the intervention group during the same periods of time. Participants of comparison group did not receive any compensation for completing the measures, and did so only motivated by their will to contribute and to being helpful. Random assignment to groups was not possible since there were not enough mothers interested in participating in the program.

The intervention group participated in an 8-week mindfulness-based program for mothers, which was an adaptation of the Mindfulness-Based Stress Reduction (MBSR) program developed by Kabat-Zinn (1990). As the MBSR, the program included weekly 2-h sessions where mothers learned and practiced mindfulness meditation, mindful yoga and shared their experiences in group discussions. Additionally, weekly homework was assigned, with a suggested daily dedication time of 30–40 min for practicing learned exercises at home. There were also exercises specifically oriented toward promoting mindful parenting and increasing awareness of patterns of parent–child interactions, whether positive or dysfunctional. For instance, one of MBSR’s homework activities consists on choosing a daily activity (e.g., brushing teeth) and intentionally direct attention to feelings, body sensations, thoughts, etc., during the activity. In this adaptation of MBSR, participants were instead asked to choose a daily activity they usually do in interaction with their children and do the same mindful exercise.

### Measures

#### Sociodemographic Questionnaire

The participants completed a questionnaire regarding contact and sociodemographic information, including the following aspects: date of birth, occupation, marital status, level of education, average level of income, number of children and their ages, level of education of the father of their preschool child, relationship of their preschool child with their father, and members of the family group (living in the same house).

#### IM-P Scale

This is a self-report questionnaire in Likert scale, developed by Duncan (2007), which evaluates mindfulness in parenting, or the extension of mindfulness to the domain of parent–child interactions. Scores range from 1 to 5. The original scale has five subscales: *Listening with full attention*; Emotional *awareness of self and child*; *Self-regulation in the parenting relationship*; *Non-judgmental acceptance of self and child*; and *Compassion for self and child* (Duncan et al., 2009). In this study, we used an adapted version of the IM-P for a sample of Chilean mothers. Factor analysis suggested a four-factor structure within the sample studied (Corthorn et al., 2015, unpublished). This IM-P version includes the following subscales: Listening with full attention, Self-regulation in the parenting relationship, Non-judgmental acceptance of self, and Empathy and acceptance for the child.

With regard to internal consistency, the reliability of this 27-item Chilean version of the IM-P was very good (α = 0.91). Internal consistencies for the new subscales were α = 0.81 for Non-judgmental acceptance for Self; α = 0.86 for Listening with Full Attention; α = 0.86 for Self-Regulation in Parenting Relationship; and α = 0.75 for Empathy and Acceptance for the Child (Corthorn et al., 2015, unpublished). Previous studies presented adequate reliability of the original IM-P scale and preliminary convergent and discriminant validity in relation to mindfulness and other parenting constructs have been demonstrated (Coatsworth et al., 2010).

#### Five-Facet Mindfulness Questionnaire (FFMQ)

The FFMQ is a 39-item measure that assesses five mindfulness domains (Baer et al., 2006). Scores range from 1 = *Never or rarely* *true* to 5 = *Very often or always true*, where higher scores reflect more mindfulness in the five aspects. The subscale Observing (α = 0.78) measures the tendency to notice or to attend to internal and external experiences, such as emotions, cognitions, sights, or smells. Describing (α = 0.90) measures the tendency to verbally describe and label these experiences. Acting with awareness (α = 0.87) refers to bringing full awareness to the current activity or experience. Non-judging (α = 0.82) refers to a non-evaluative stance toward inner experiences. Non-reactivity (α = 0.79) measures the tendency to allow thoughts and feelings to come and go, without getting carried away by them. The construct validity of FFMQ has been assessed in meditating and non-meditating samples (Baer et al., 2006, 2008). In Chile, good construct reliability was found as well. α = 0.91 for the general scale. Scores ranged from 0.75 to 0.88 in Cronbach’s alpha for the five subscales (Solari, 2010).

#### Parenting Stress Index-Short Form

The self-report questionnaire uses a Likert scale, with scores ranging from 1 to 5, developed by Abidin (1995). It measures parents’ or caregivers’ levels of stress associated with their role as parents. The abbreviated form used in this study includes 36 items, divided into the following three subscales*: Parental Distress (PD), Parent–Child Dysfunctional Interaction (P-CDI)*, and *Difficult Child (DC)*. The sum of these subscales generates a final global score named Total Stress, which refers to the level of stress that the caregiver perceives regarding his/her role. Validity studies have been conducted in several cultures. A sample of 800 American families indicated reliability with test–retest methodology. The coefficients obtained were 0.84 (total score), 0.85 (PD), 0.78 (DC), and 0.68 (P-CDI), and Cronbach’s alpha values of 0.91 (total score), 0.87 (PD), 0.85 (DC), and 0.80 (P-DCI). Validity levels show correlations between 0.73 and 0.95 (Abidin, 1995). Reliability has not been validated in Chile, but this measure has been applied in different cultures (e.g., Chinese, Italian, Portuguese, French, and Latin-American) and is widely accepted. Cronbach’s alpha values obtained within the present study’s pre-intervention sample were 0.89 (total score), 0.91 (PD), 0.63 (P-CDI), 0.83 (DC). Post-intervention Cronbach’s alpha values were 0.83 (total score), 0.74 (PD), 0.62 (P-CDI), 0.80 (DC).

#### DASS-21

The Depression Anxiety Stress Scale 21 (DASS-21) is a short form of Lovibond and Lovibond’s (1995) 42-item self-report measure of DASS. A four-point severity scale measures the extent to which each state has been experienced over the past week. The DASS-21 consists of three 7-item self-report scales taken from the full version of the DASS. DASS-21 was translated and adapted in Chile by Vinet et al. (2008) and modified by Román (2010). Psychometric studies support its use in the Chilean population (Antúnez and Vinet, 2012). Within the present study’s pre-intervention sample Cronbach’s alpha values were 0.77 for Stress, 0.65 for Anxiety, and 0.86 for Depression. Within post-intervention sample values were 0.72 for Stress, 0.77 for Anxiety, and 0.76 for Depression.

### Statistical Analyses

First, descriptive analyses regarding initial levels of the study variables were performed, including evaluation of mean differences between the intervention and the comparison group.

Second, a mixed ANOVA 2 × 3 was performed for each variable in the study, with a between factor given by group (intervention × comparison) and a within factor given by the three measurements of the dependent variables: before and after the intervention, and a subsequent 2-month follow-up.

Finally, when *omnibus* mixed ANOVA was significant, specific simple effects were evaluated through mixed ANOVA 2 × 2, to determine whether the change occurred between pre- and post-intervention measures, or afterward, between post-intervention and follow-up.

## Results

### Descriptive Analyses

The participants (control and intervention group) presented initial levels of Depression (*M* = 3.07, *SD* = 3.28), Anxiety (*M* = 2.47, *SD* = 2.33), and Stress (*M* = 5.77, *SD* = 3.11) within normal levels according to DASS-21. The PSI-SF total and subscales means were at adequate levels according to PSI-SF interpretation guidelines (Abidin, 1995): PSI total (*M* = 74.02, *SD* = 18.61), Parental Distress (*M* = 28.55, *SD* = 10.34), Parent–Child Dysfunctional Interaction (*M* = 18.94, *SD* = 4.53), and Difficult Child (*M* = 26.54, *SD* = 8.03).

An independent sample *t*-test was conducted to compare initial intervention and comparison group means. Both presented statistical equivalence regarding sociodemographic characteristics (mother’s age, marital status and educational level, child’s age, father’s educational level, family income, number of children) and levels of anxiety and depression. The intervention group presented lower levels of mindfulness, mindful parenting, and higher levels of stress, and parental stress. See **Table 1** for means, standard deviation, *t*-values, and level of significance. The difference in initial measures was probably due to self-selection: stressed mothers sought the program and therefore enrolled in greater numbers than less stressed mothers. Because mindfulness is associated with stress levels, this was also reflected. It is important to note that the repeated measures in the ANOVA analysis considered the differences between the gain scores of each group (within subjects time × group interaction effect), not the post-treatment group differences (between subject post-intervention group effect). This approach is recommended particularly in cases of self-selection because analyzing post-treatment between group effects may result in false negative results, whereas differences in gain scores between groups more accurately reflect change (Rogosa, 1988; Maris, 1998).

**Table 1:** Pre-intervention means and standard deviations.

Variable	Group	*M*	*SD*	*t*	*N*
Age	Intervention	34.62	5.53	-1.196	21
	Comparison	36.50	4.78		22
Marital status	Intervention	2.29	.96	0.048	21
	Comparison	2.27	.83		22
Educational level	Intervention	4.48	.68	-0.888	21
	Comparison	4.64	.49		22
Father’s educational level	Intervention	4.29	.78	-0.922	21
	Comparison	4.50	.74		22
Child’s age	Intervention	2.90	.89	-0.334	21
	Comparison	3.00	.98		22
Number of children	Intervention	1.76	.89	-0.043	21
	Comparison	1.77	.75		22
Income	Intervention	3.95	1.28	-1.235	21
	Comparison	4.41	1.14		22
Mindful parenting	Intervention	91.00	10.01	-3.586**	21
	Comparison	102.27	10.57		22
Mindfulness	Intervention	114.14	17.92	-2.987**	21
	Comparison	131.46	19.98		22
Depression	Intervention	4.05	3.65	1.973	21
	Comparison	2.14	2.64		22
Anxiety	Intervention	2.86	2.46	1.078	21
	Comparison	2.09	2.20		22
Stress	Intervention	7.43	3.11	3.985**	21
	Comparison	4.18	2.17		22
Parental stress	Intervention	83.42	19.14	3.692**	21
	Comparison	65.04	13.09		22


*^∗^Significant at *p* < 0.05; ^∗∗^significant at *p* < 0.01*.

### Mixed ANOVA

#### Parental Stress (PSI-SF)

ANOVA 3 × 2 test indicated a statistically significant decrease in the PSI global score in the intervention group, compared to the comparison group [*F*(2,70) = 15.170, *p* < 0.001], with a medium effect size *(d* = 0.657). Mauchly’s Test indicated that the assumption of sphericity had not been violated [χ^2^(2) = 3.293, *p* = 0.193]. *Post hoc* ANOVA 2 × 2 tests revealed that the significant effects occurred between time 1 and time 2 measures [*F*(1,41) = 15.408, *p* < 0.001]. No statistically significant changes occurred between time 2 and time 3, meaning that participants maintained their scores.

**FIGURE 1:**
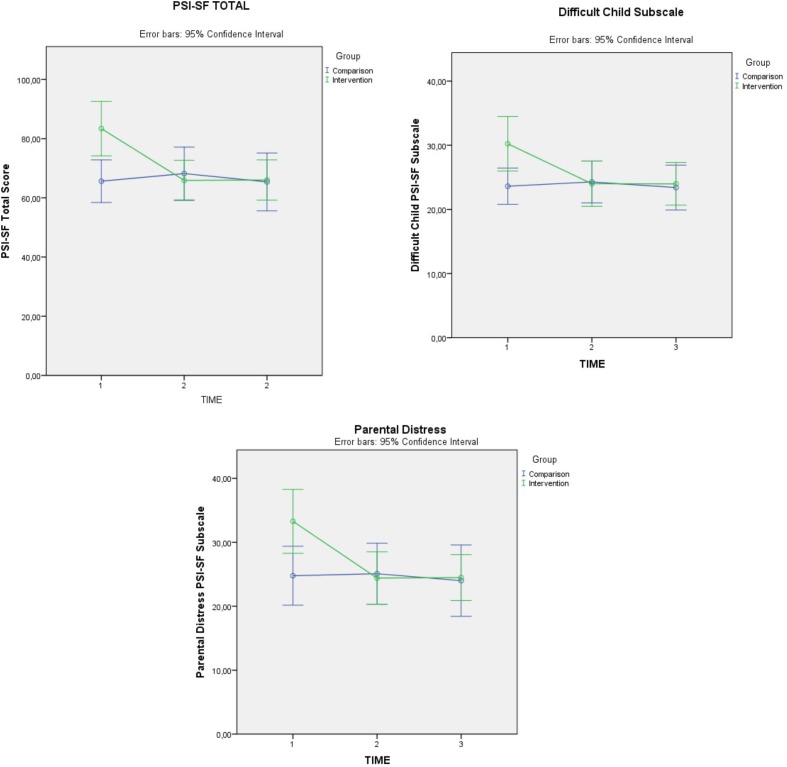
PSI-SF total score, parental distress and difficult child marginal pre, post, and follow-up means.

With regard to the subscales, there was a statistically significant decrease for two of the three subscales: “Parental Distress” [*F*(2,70) = 9.007, *p* < 0.001] and “Difficult Child” [*F*(2,72) = 6.553, *p* = 0.002], with medium (*d* = 0.508) and small effects size (*d* = 0.427), respectively. For both subscales Mauchly’s Test of Sphericity indicated that the assumption of sphericity had not been violated [χ^2^(2) = 5.704, *p* = 0.058 for Parental Distress; χ^2^(2) = 1.021, *p* = 0.600 for Difficult Child]. Additionally, *post hoc* ANOVA 2 × 2 indicated that the significant results for both subscales occurred between time 1 and time 2 [*F*(1,41) = 12.249, *p* = 0.001 for Parental Distress; *F*(1,41) = 8.855, *p* = 0.005 for Difficult Child], with scores between time 2 and time 3 showing no significant change. **Figure 1** illustrates the control and intervention groups’ mean trajectories in time, for the variables that presented significant change. There was no significant effect for “Parent–Child Dysfunctional Interaction.” See **Table 2** for means and standard deviations of the PSI-SF total score and subscales for the three measurements.

**Table 2:** DASS-21 means and standard deviation scores.

DASS-21		Pre-intervention			Post-intervention			Follow up		
		*M*	*SD*	*N*	*M*	*SD*	*N*	*M*	*SD*	*N*
Depression	Intervention	4.25	3.63	20	1.80	1.85	20	2.25	2.55	20
	Comparison	2.24	2.99	17	2.12	2.69	17	2.41	2.15	17
Anxiety	Intervention	3.00	2.43	20	1.85	2.13	20	1.50	2.16	20
	Comparison	1.88	2.21	17	1.00	1.90	17	2.59	4.87	17
Stress	Intervention	7.60	3.09	20	4.05	2.91	20	4.33	3.47	20
	Comparison	4.24	2.39	17	3.82	2.13	17	3.94	4.19	17


#### Depression, Anxiety, and Stress (DASS-21)

**FIGURE 2:**
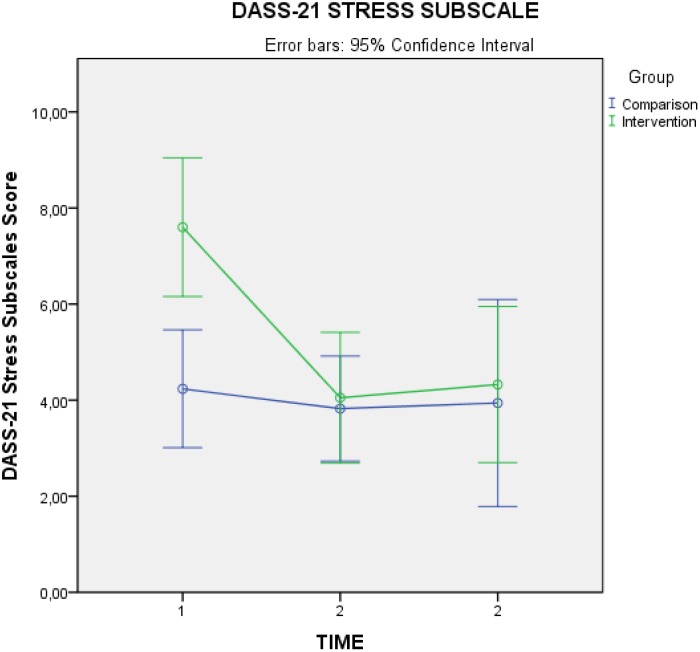
DASS-21 stress subscale marginal pre, post, and follow-up means.

There was a statistically significant decrease in intervention group level of stress, compared to the comparison group [*F*(2,70) = 5.378, *p* = 0.007], with a medium effect size considering Cohen’s *d* (*d* = 0.638). Mauchly’s Test of Sphericity indicated that the assumption of sphericity had not been violated, χ^2^(2) = 5.274, *p* = 0.072. As with the previous measures, the *post hoc* ANOVA 2 × 2 tests indicated that significant effects occurred between time 1 and time 2 [*F*(1,41) = 11.716, *p* = 0.001], with no significant change observed in the follow-up measure. There were no statistically significant effects for depression and anxiety. See **Table 3** for means and standard deviations and **Figure 2** for graphic representation of mean changes in stress over time.

**Table 3:** IM-P means and standard deviation scores.

IM-P		Pre-intervention			Post-intervention			Follow up		
		*M*	*SD*	*N*	*M*	*SD*	*N*	*M*	*SD*	*N*
Total score	Intervention	91.3	10.17	20	103.65	9.06	20	105.35	8.93	20
	Comparison	102.05	10.19	18	102.94	10.21	18	103.33	11.57	18
Non-judgmental acceptance	Intervention	16.35	3.84	20	21.00	3.63	20	21.95	3.52	20
	Comparison	19.00	4.27	18	18.89	3.98	18	19.28	5.14	18
Listening with full attention	Intervention	16.00	2.43	20	17.85	2.46	20	18.10	2.38	20
	Comparison	18.56	2.66	18	18.78	2.73	18	19.11	3.05	18
Self-regulation in parenting	Intervention	21.50	3.55	20	24.65	2.56	20	24.55	2.44	20
	Comparison	23.77	4.48	18	24.22	3.69	18	24.44	3.76	18
Empathy and acceptance	Intervention	37.45	3.78	20	40.15	3.84	20	40.75	3.58	20
	Comparison	40.72	3.06	18	41.05	3.31	18	40.50	3.52	18


#### Mindful Parenting (IM-P)

**FIGURE 3:**
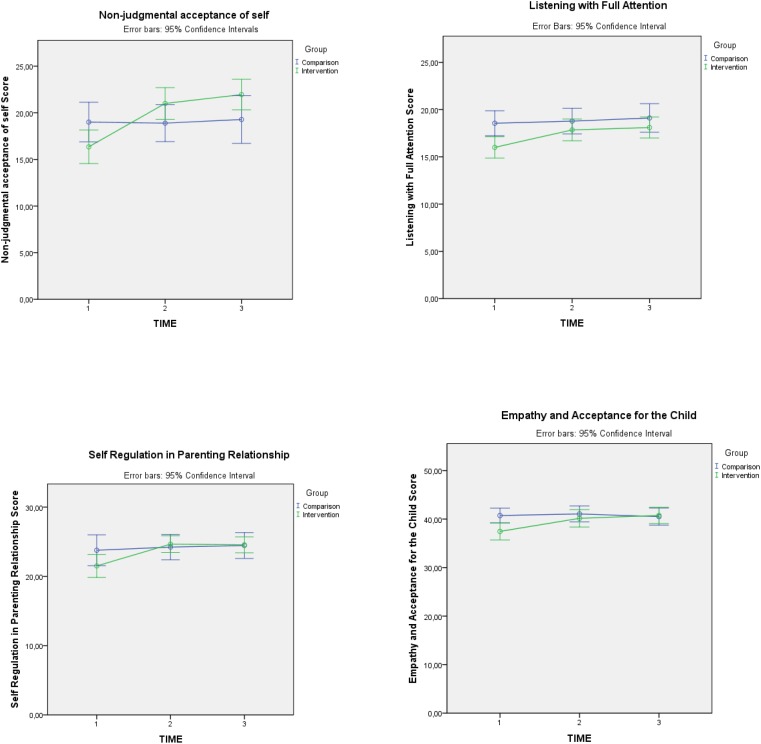
IM-P subscales marginal pre, post, and follow-up means.

All of the IM-P subscale scores of the intervention group presented a statistically significant increase compared to the comparison group, with medium to small effects sizes: *Non-judgmental acceptance of self as a mother* [*F*(2,72) = 10.034, *p* < 0.001, *d* = 0.528]; *Listening with Full Attention* [*F*(2,72) = 3.841, *p* = 0.033, *d* = 0.326]; *Self-regulation in parenting relationship* [*F*(2,72) = 4.277, *p* = 0.025, *d* = 0.344]; and *Empathy and acceptance for the child* [*F*(2,72) = 9.617, *p* < 0.001, *d* = 0.517]. The assumption of sphericity was not met for Listening with full attention [χ^2^(2) = 6.993, *p* = 0.030], Self-regulation in the parenting relationship [χ^2^(2) = 13.156, *p* = 0.001] and Empathy and acceptance for the child [χ^2^(2) = 7.013, *p* = 0.030], thus the *p-*values previously reported for these subscales correspond to Greenhouse–Geisser correction when epsilon was smaller than 0.75 (Listening with Full Attention) and Huynh–Feldt correction when epsilon was larger than 0.75 (Self-regulation in the parenting relationship and Empathy and acceptance for the child). See **Table 4** for means and standard deviations and **Figure 3** for a graphic representation of mean changes in IM-P total scores and subscales.

**Table 4:** FFMQ means and standard deviation scores.

FFMQ		Pre-intervention			Post-intervention			Follow up		
		*M*	*SD*	*N*	*M*	*SD*	*N*	*M*	*SD*	*N*
Total score	Intervention	113.05	17.65	20	132.65	18.42	20	135.54	16.34	20
	Comparison	131.88	20.96	17	138.55	18.54	17	137.70	18.91	17
Observe	Intervention	24.15	6.34	20	28.55	5.56	20	28.05	5.74	20
	Comparison	27.44	5.98	18	26.67	6.09	18	27.61	5.86	18
Describe	Intervention	23.80	5.40	20	27.65	6.46	20	27.95	6.65	20
	Comparison	29.11	4.86	18	30.61	5.12	18	30.70	5.56	18
Act with awareness	Intervention	21.80	5.85	20	25.80	5.42	20	27.74	5.73	20
	Comparison	26.83	6.80	18	29.37	5.56	18	28.67	5.78	18
Non-judge	Intervention	22.25	4.87	20	26.90	4.30	20	28.75	5.57	20
	Comparison	25.11	5.36	18	27.56	6.00	18	26.96	7.11	18
Non-react	Intervention	21.05	3.14	20	23.75	3.58	20	23.05	3.40	20
	Comparison	22.35	4.94	17	23.27	4.96	17	22.51	6.49	17


Again, *post hoc* ANOVA 2 × 2 tests indicated that significant effects occurred between time 1 and time 2 measures, whereas no significant increase or decrease was observed between time 2 and time 3. Pre–post significant effects for each IM-P subscale and the total score were as follows: Non-judgmental acceptance of self as a mother [*F*(1,41) = 11.399, *p* = 0.002], Listening with Full Attention [*F*(1,41) = 11.961, *p* = 0.019], Self-Regulation in the Parenting Relationship [*F*(1,41) = 8.461, *p* = 0.006], Empathy and acceptance for the child [*F*(1,41) = 9.423, *p* = 0.004].

#### FFMQ Mindfulness

There was a statistically significant increase in the subscales “Observe” [*F*(2,72) = 6.384, *p* = 0.005] and “Non-judge” [*F*(2,72) = 4.161, *p* = 0.023], all with small size effects, *d* = 0.42, *d* = 0.42, and *d* = 0.34, respectively. The *p-*values reported correspond with the Huynh–Feldt correction because sphericity assumption was not met and the epsilon value was higher than 0.75 [χ^2^(2) = 12.899, *p* = 0.002 for FFMQ; χ^2^(2) = 11.483, *p* = 0.003 for “Observe”; χ^2^(2) = 6.345, *p* = 0.042 for “Non-judge”]. As expected, the subscales “Describe” and “Act with Awareness” did not indicate any significant changes, and, contrary to what was expected, the “Non-react” subscale did not indicate any change either. It was not possible to statistically verify whether “Act with Awareness” subscales presented a significant increase in initially low scores and a decrease in high scores because the sample size for low score and high score cases was too small. However, it could be observed that initial low scores did increase, but it was not possible to determine if this was statistically significant when compared with the comparison group. Initial high scores did not decrease, but maintained upper levels in the second and third measurement.

**FIGURE 4:**
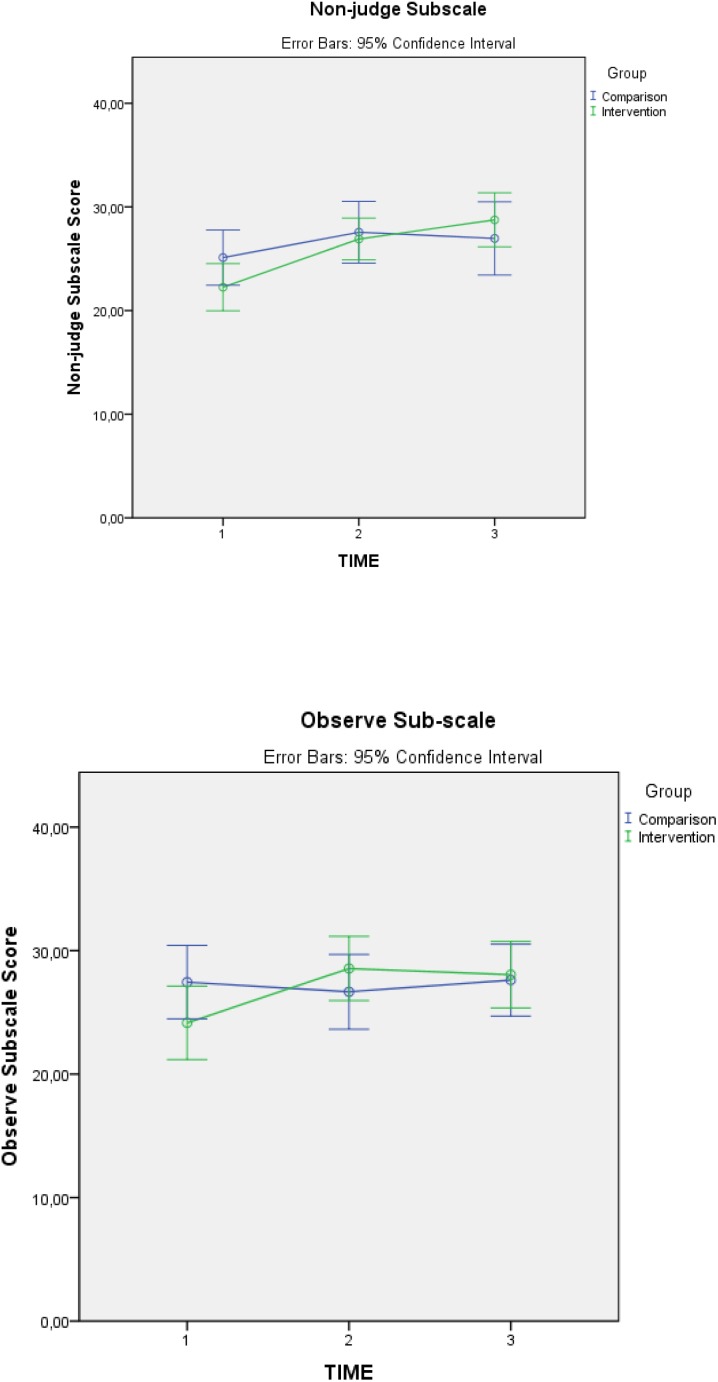
FFMQ observe and non-judge subscales marginal pre, post, and follow-up means.

Mixed ANOVA 2 × 2 *post hoc* tests indicated that the changes occurred between time 1 and time 2 for the “Observe” subscale [*F*(1,41) = 10.188, *p* = 0.003], with no significant change between time 2 and time 3, whereas the “Non-judge” subscale did not significantly change compared to the comparison group between time 1 and time 2 or between time 2 and time 3. A significant increase in the “Non-judge” subscale in the intervention group compared to the comparison group occurred between time 1 and time 3 [*F*(1,36) = 7.192, *p* = 0.011]. See **Table 4** for means and standard deviations and **Figure 4** for graphic representation of mean changes of “Non-judge” and “Observe” subscales.

## Conclusion and Discussion

Nearly all of the study hypotheses were confirmed. As predicted, the intervention group showed a significant reduction in general and parental stress and an increase in mindful parenting and general mindfulness variables when compared with the comparison group. Effect sizes ranged from small to medium, with the highest Cohen’s *d* in stress (general and parental) and mindful parenting. This findings were coherent with previous studies (Singh et al., 2007; Vieten and Astin, 2008; Duncan and Bardacke, 2010; Bögels et al., 2013; Perez-Blasco et al., 2013) and add more evidence regarding the importance of mindfulness interventions. This is particularly relevant considering the implications of parental stress in parent–child interactions and parenting (Webster-Stratton, 1990; Creasey and Jarvis, 1994; Sidebothan, 2001; Bonds et al., 2002).

There was also a significant effect of mindfulness “Observe” and “Non-judge” subscales and a statistically significant increase in mindful parenting variables, including all subscales: Listening with full attention, Self-regulation in the parenting relationship, Non-judgmental acceptance of self, and Empathy and acceptance of the child. Each of them promotes better parenting. Listening with full attention is related to the central mindfulness aspect of clear attention and receptive awareness in moment-to-moment experiences (“Acting with awareness”). Duncan et al. (2009) noted that their model of mindful parenting “pairs full attention with listening because it is by directing their full attention to their child that parents convey that they are truly listening to their child” (p. 259). Empathy and acceptance of the child implies a parent’s desire to meet appropriate child needs and to comfort the child when they are feeling distress. Non-judgmental acceptance of self and child and self-regulation in parenting interactions are more specific aspects of “non-judging” and “non-reacting,” applied during parent–child interactions. For parents in this study, the mindfulness ability of non-judgment seemed to be particularly enhanced. Mindful parenting dimensions that involve non-judgment, that is, *Non-judgmental acceptance of self as a mother* and *Empathy and acceptance for the child*, were the ones with higher size effects. Additionally, “Non-judge” was among the two mindfulness aspects that were statistically significant. As noted in the introduction, non-judgment seems to be a particularly important aspect of mindfulness regarding reduction of stress, anxiety and depression and other mental health-related variables in the general population and in mothers (Baer et al., 2006; Cash and Whittingham, 2010; Corthorn and Milicic, 2015). In particular, the IM-P subscales *Non-judgmental acceptance of self as a mother* and *Empathy and acceptance for the child* have been found to predict parental stress and general stress in mothers (Corthorn and Milicic, 2015). It is important to take in consideration that these subscales included items from original IM-P subscales “Compassion for Self and Child.” Non-judgment can be thought of as a form of expression of compassion or loving-kindness toward one-self or others, allowing that space of acceptance to whatever arises. Attitudes of non-judgment and gentleness promoted in mindfulness-based interventions, including the one in the present study, seem reflected by self-compassion, defined as the recognition and clear seeing of one’s own suffering and the desire to ameliorate it with kindness, recognizing our shared human condition as flawed and fragile. Being kind and compassionate toward oneself involves being less self-critical and reducing negative self-judgment (Neff, 2011). These findings suggest that programs directed toward mothers should give special attention to fostering the cultivation of non-judgment and compassion. Additionally, these programs should carefully avoid the increase in self-blame and guilt, which can be an unwanted effect of parent training programs where participants may feel bad about their current and past parenting practices when comparing them with the ones taught in the program.

In all cases, except the “Non-judge” mindfulness dimension, significant change was observed between pre- and post-test measures. Follow-up measures indicated no significant change since post-intervention, which means that the effects of the intervention were maintained after 2 months. As mentioned, a significant increase in “Non-judge” occurred between pre-intervention and follow-up, suggesting that it may take more time for this dimension to indicate change. The “Non-judgmental acceptance of self as a mother” dimension did indicate change in the post-intervention measure.

It is interesting to note that even though the program did not highly differed from MBSR, the few adaptations apparently made a difference regarding its focus in parenting. The size of the effects were higher on mindful parenting than on mindfulness and while all mindful parenting aspects presented significant change, only two mindfulness subscales did too. The formal practice was the same as included on MBSR curriculum, mainly body-scan, mindful yoga, and sitting meditation. What differed was the focus of the “informal mindfulness practice.” Besides, the initial motivation that decided the mothers to participate was probably different from typical MBSR’s participants. Many of them explicitly said that they were doing this “for their children.” So, from the beginning their main intention was to learn mindfulness in order to improve their parenting, which may have impacted the results. This result suggests the importance of addressing mindful parenting as a specific and differentiated application of mindfulness and of mindfulness-based programs for parents.

### Limitations and Future Research

Although the present study compared the intervention group with the comparison group, it was not possible to randomly assign participation to either group. A waiting list approach was not viable because there were not enough mothers interested in participating in the program. For this reason, there was an initial difference between the groups in some of the study variables. The data analysis took into account differences in the gain scores of each group and not the post-treatment group differences, which is an approach that more accurately reflects change in cases of self-selection (Rogosa, 1988; Maris, 1998).

Furthermore, the data were gathered through self-report questionnaires, so definite conclusions regarding actual change in mother–child interactions were not ascertainable. Nevertheless, a recent study by Duncan et al. (2015) provides preliminary evidence about a link between observed parent–child interactions and mindful parenting measured through the same self-report measure used in this study.

Future research should study the effects of mindful parenting on other relevant parenting variables such as self-competence and utilize observational studies of mother–child interactions and child outcomes. It would also be interesting to inquire whether non-judgmental acceptance toward self and the child mediate the effects of mindfulness-based intervention on mothers’ general and parental stress.

## Ethics Statement

All procedures performed in this study were approved by the ethical committee of the School of Psychology of the *Pontificia Universidad Católica de Chile* (Catholic University of Chile) and in accordance with the 1964 Helsinki declaration and its later amendments. Informed consent was obtained individually from all of the participants included in the study.

## Author Contributions

CC participated on the conception and design of the study, analysis and interpretation of data, as well as drafting the manuscript; and agrees to be accountable for all aspects of the present work.

## Conflict of Interest Statement

The author declares that the research was conducted in the absence of any commercial or financial relationships that could be construed as a potential conflict of interest.
